# A Family-Based Mental Health Navigator Intervention for Youth in the Child Welfare System: Protocol for a Randomized Controlled Trial

**DOI:** 10.2196/49999

**Published:** 2023-09-12

**Authors:** Marina Tolou-Shams, Megan Ramaiya, Jannet Lara Salas, Ifunanya Ezimora, Martha Shumway, Jill Duerr Berrick, Adrian Aguilera, Brian Borsari, Emily Dauria, Naomi Friedling, Crystal Holmes, Adam Grandi

**Affiliations:** 1 Department of Psychiatry and Behavioral Sciences University of California, San Francisco San Francisco, CA United States; 2 Berkeley Social Welfare University of California at Berkeley Berkeley, CA United States; 3 San Francisco Veteran Affairs Medical Center San Francisco, CA United States; 4 School of Public Health University of Pittsburgh Pittsburgh, PA United States; 5 Foster Care Mental Health San Francisco, CA United States

**Keywords:** randomized clinical trial, foster care, child welfare–involved youth, navigator interventions, digital health technology, implementation science, community engagement

## Abstract

**Background:**

Youth in the child welfare system (child welfare–involved [CWI] youth) have high documented rates of mental health symptoms and experience significant disparities in mental health care services access and engagement. Adolescence is a developmental stage that confers increased likelihood of experiencing mental health symptoms and the emergence of disorders that can persist into adulthood. Despite a high documented need for evidence-based mental health services for CWI youth, coordination between child welfare and mental health service systems to increase access to care remains inadequate, and engagement in mental health services is low. Navigator models developed in the health care field to address challenges of service access, fragmentation, and continuity that affect the quality of care provide a promising approach to increase linkage to, and engagement in, mental health services for CWI youth. However, at present, there is no empirically supported mental health navigator model to address the unique and complex mental health needs of CWI youth and their families.

**Objective:**

Using a randomized controlled trial, this study aims to develop and test a foster care family navigator (FCFN) model to improve mental health service outcomes for CWI adolescents (aged 12-17 years).

**Methods:**

The navigator model leverages an in-person navigator and use of adjunctive digital health technology to engage with, and improve, care coordination, tracking, and monitoring of mental health service needs for CWI youth and families. In total, 80 caregiver-youth dyads will be randomized to receive either the FCFN intervention or standard of care (clinical case management services): 40 (50%) to FCFN and 40 (50%) to control. Qualitative exit interviews will inform the feasibility and acceptability of the services received during the 6-month period. The primary trial outcomes are mental health treatment initiation and engagement. Other pre- and postservice outcomes, such as proportion screened and time to screening, will also be evaluated. We hypothesize that youth receiving the FCFN intervention will have higher rates of mental health treatment initiation and engagement than youth receiving standard of care.

**Results:**

We propose enrollment of 80 dyads by March 2024, final data collection by September 2024, and the publication of main findings in March 2025. After final data analysis and writing of the results, the resulting manuscripts will be submitted to journals for dissemination.

**Conclusions:**

This study will be the first to produce empirically driven conclusions and recommendations for implementing a family mental health navigation model for CWI youth with long-standing and unaddressed disparities in behavioral health services access. The study findings have potential to have large-scale trial applicability and be feasible and acceptable for eventual system implementation and adoption.

**Trial Registration:**

ClinicalTrials.gov NCT04506437; https://www.clinicaltrials.gov/study/NCT04506437

**International Registered Report Identifier (IRRID):**

DERR1-10.2196/49999

## Introduction

### Background

After a decade-long decline in the number of youth in placement outside of the home (ie, foster care) in the United States, the foster care caseload in most states rose between 2014 and 2019. The current foster care caseload exceeds 400,000 in the United States [1], with another approximately 165,000 youth receiving court-mandated services within their own homes [2]. Youth in out-of-home or foster care placement and those remaining in the home but are child welfare system and court involved are collectively referred to as *child welfare–involved (CWI)* and are considered some of the most underserved with respect to behavioral health care access in the United States. The COVID-19 pandemic has further increased the number of youth entering the child welfare system owing to having lost parents or caregivers from COVID-19–related death [3].

Rates of mental health symptoms and diagnoses in CWI youth are high, particularly among adolescents. The vast majority are referred to child welfare agencies because of child abuse or neglect [4], and, as a result of trauma experienced in their home of origin, the majority display mental health or behavioral health challenges [5]. According to the National Survey of Child and Adolescent Well-Being, much more than half of a national sample of youth in out-of-home care display behavioral or social competency problems [6], and studies that include smaller samples or regional samples suggest similar results [7,8]. Among 815 CWI youths aged 12 to 17 years, 43% reported at least 1 mental health concern, including attention-deficit/hyperactivity disorder (ADHD; 19%), suicidality (14%), anxiety (14%), depression (9%), and co-occurring substance use (23%) [9]. Youth in out-of-home care report higher rates of mental health problems than peers of a similar age [10,11]. Depending on the study, between 35% and 85% of all youth in out-of-home care experience a mental health condition [12], and youth in out-of-home care are 3 times more likely to be prescribed psychotropic medications for their mental health conditions [13].

Adolescence is a critical developmental period for the assessment, diagnosis, and treatment of mental health symptoms. Vulnerability to mental health disorders increases significantly in adolescence, and the emergence of disorders during this period (eg, early psychosis) increases the likelihood of persistence into adulthood [14]. Rates of depression escalate in adolescence, particularly among girls with extensive trauma histories (such as CWI youth) who are at substantially higher risk for poor mental health, substance use, and legal outcomes. Adolescence also encompasses unique factors associated with the transition to young adulthood (eg, for those aged 16-17 years), making it a highly vulnerable developmental stage for CWI youth [9]. Adolescents who are placed in out-of-home care face unique risks because chronic placement instability is the norm rather than the exception, particularly for older adolescents [15-17]. Studies demonstrate that youth with mental health difficulties are more likely to be moved from one placement to another, and these moves create or exacerbate mental health concerns [18,19]. These concerns are exacerbated for racial and ethnic minoritized youth [20] and reify ongoing and long-standing behavioral health disparities among CWI youth. Identifying adolescent mental health needs, matching these needs to appropriate services, and providing supports for mental health care access and linkage have great potential to resolve or offset challenges manifesting in adulthood.

Despite high need for evidence-based mental health services for CWI youth, ample evidence suggests that coordination between child welfare and mental health service systems is inadequate. Access to care for CWI youth is hampered by multiple system-level barriers, such as frequent change of placement as well as underreporting of mental health problems by foster parents, social workers, and physicians [13]. National survey data (n=3803) highlight a substantial gap between the identified need for and use of mental health services in child welfare populations, particularly among older youth: 66% of those aged 11 to 14 years have clinically significant mental health needs, yet only 26% use mental health services [21]. Data local to the site of the proposed project demonstrate critical gaps in mental health screening and full assessment, with only 28% of all youth assessed within 2 months of case opening, 45% by 3 months, and 69% by 6 months. Furthermore, by 12 months, 11% of youth had their case closed before ever receiving a mental health assessment [22].

The public health impact of gaps in mental health services delivery among CWI youth is significant. Untreated mental health concerns among CWI youth persist when they leave the out-of-home care system and transition to independence [23]. CWI youth are also likely to have dual (or *crossover*) involvement with the juvenile legal system [24], which can further exacerbate unmet services needs and disparities within this population. Persistent mental health concerns in this population can also contribute to placement instability [25] and re-entry into the child welfare and juvenile legal systems [26], which both compound mental health difficulties and strain public health systems and economies. Unaddressed mental health services needs also perpetuate racial and ethnic disparities in services access, engagement, and quality in this population, as well as perpetuate the root causes of these disparities (eg, systemic racism and discrimination), which have notable public health consequences. Data local to the site of the proposed project mirror national statistics, documenting the alarming overrepresentation of African American and Latinx youth in the child welfare system [21]: 36% of CWI youth aged 12 to 17 years are African American compared with 6.1% of the entire county population.

Navigator models (ie, the use of a navigator to support access to, and transition through, complex health care systems) provide a promising approach to increasing linkage to, and engagement in, mental health services for CWI youth with high rates of unaddressed mental health concerns. Navigator models have been successfully developed and deployed in other contexts (eg, cancer and HIV), with demonstrated improvements in service tailoring, linkage, and treatment retention [27]. A small number have also been developed for general adolescent populations with behavioral health needs (eg, the Family Navigation Project) [28]. However, existing mental health navigator models have not been empirically developed or tailored for system-involved (ie, child welfare or juvenile legal system) youth and families who have unique, complex multisystemic factors associated with mental health care needs and access.

Mobile health (mHealth) technology is gaining empirical support for improving adolescent mental health and behavioral health (eg, substance use) outcomes. Data suggest that SMS text messaging is a useful tool to improve youth attendance in outpatient mental health treatment [29] and can prevent adolescent substance use relapse [30]. A recent meta-analysis (n=14 studies) concluded that SMS text messaging technology is a promising substance use prevention strategy for adolescents and young adults [31], the majority of whom have access to, and regularly use, their mobile phones despite unique adolescent barriers to constant access and use (eg, service payment, continuous service, and no access while at school during the day). Our own work with community-supervised court-involved adolescents and their caregivers suggests that families and involved juvenile legal personnel and providers all heavily rely on digital and mHealth technology for communication and care coordination (eg, SMS text messaging, emailing, and social media instant messaging) [32].

Despite the promise and prevalence of mHealth technology, there is no systematic, empirically supported strategy leveraging mHealth to enhance family-based approaches to improving CWI youth mental health service use and outcomes. Studies show that only some caregivers are ready for electronic messaging support for health care [33] and that depending on caregiver race or ethnicity (eg, Latinx ethnicity), socioeconomic status (eg, low status) and age (eg, younger age), SMS text messaging may be more or less appealing as a tool for adolescent health care engagement [33,34]. Parent outreach via bidirectional SMS text messaging to enhance the uptake of adolescent vaccine and well care services improved these adolescent health outcomes [35], suggesting that a caregiver-level mHealth intervention may be effective in improving other adolescent health outcomes (eg, mental health). Caregivers of CWI youth have the additional context of juvenile legal system and child welfare system surveillance and involvement (eg, mandated youth treatment, sanctions-based approach to noncompliance, and information-sharing concerns) that might differentially affect their response to mental health navigator interventions, particularly those that incorporate mHealth for service tracking or monitoring. Given the high numbers of staff, providers, and systems that often operate separately and with minimal coordination to support CWI youth, mHealth has unique potential to improve communication between families and systems to support linkage to, and engagement in, mental health services.

Given the significant need for an empirically driven, timely, coordinated, and collaborative interagency response to addressing the mental health needs of CWI youth, the proposed project aims to develop and test an empirically derived navigator intervention that relies on interagency codevelopment and incorporates state-of-the-art mHealth technology to improve linkage to, and engagement in, mental health services among CWI youth. A randomized clinical trial (N=80 caregiver-youth dyads) comparing the navigator intervention with standard of care (clinical case management services) for CWI youth has the potential to have large-scale trial applicability and to be feasible and acceptable for eventual system implementation and adoption.

### Study Objectives

This study will be the first to develop and test a foster care family navigator (FCFN) intervention for CWI youth that has the potential to significantly improve adolescent mental health disparities, particularly for racial and ethnic minoritized youth. The primary goal of the study is to assess the feasibility, acceptability, and preliminary impact of the FCFN intervention on the mental health services cascade of care (eg, screening, assessment, and treatment initiation) outcomes. Using a randomized controlled trial (RCT) design, we will randomize 80 families to receive either the FCFN intervention or standard of care (clinical case management services): 40 (50%) to FCFN and 40 (50%) to control. Qualitative exit interviews will inform the feasibility and acceptability of the services received during the 6-month period. Primary trial outcomes are mental health treatment initiation and engagement. Other pre- and postservice outcomes, such as proportion screened and time to screening, will also be evaluated.

### Juvenile Justice Behavioral Health Services Cascade

**Figure 1:**
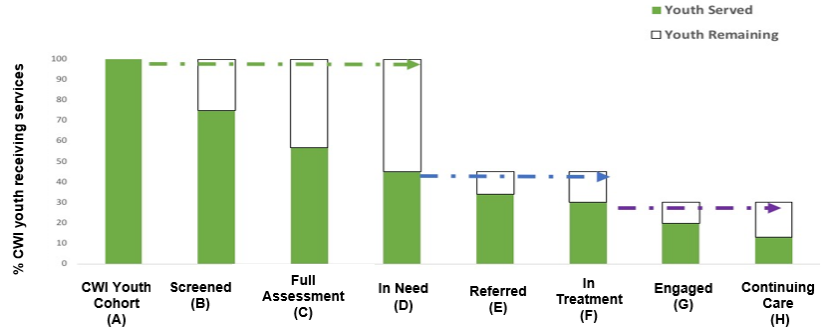
Example of juvenile justice behavioral health services cascade adapted for child welfare–involved (CWI) youth.

The juvenile justice behavioral health services cascade, hereinafter referred to as the *cascade*, was recently developed (adapted from the HIV cascade of care models) [36] as part of the Juvenile Justice Translational Research on Interventions for Adolescents in the Legal System (JJ-TRIALS) study, a National Institute on Drug Abuse–funded, large-scale, multisite research and implementation science collaborative. JJ-TRIALS developed the *cascade* as a unifying framework for juvenile legal system and behavioral health partners to collectively (1) identify system-level substance use service strengths and needs along the continuum of care and (2) codevelop and test interventions to improve adolescent substance use service linkage and outcomes along the *cascade*. The *cascade* framework can also inform multiple interrelated research goals and be easily adapted for empirical application and testing with other youth populations (eg, foster care youth), systems (eg, child welfare system) and health (eg, mental health) outcomes [37] as in the proposed study. Figure 1 presents a hypothetical adapted *cascade* framework for CWI adolescent populations using the same cascade of care steps but modifying the involved systems (eg, from juvenile legal system to child welfare system). Table 1 presents step definitions (developed by the JJ-TRIALS group) adapted to CWI youth and system processes.

**Table 1:** Behavioral health services cascade definitions adapted for child welfare–involved youth and the foster care mental health (FCMH) system.

Step	Operational definition	Calculated proportion (based on step)
A. Child welfare referrals	Total number of referrals to FCMH over a designated period of time, less any youth already in treatment at that time	N/A^a^
B. Screened	Subset of FCMH referrals (A) with a screening date	B/A
C. Full clinical assessment	Subset of FCMH referrals (A) with a full clinical assessment (includes if follow-up to screening or other clinical assessment)	C/A
D. In need	Subset of FCMH referrals (A) with a need for mental health treatment based on screener, clinical assessment, or other sources of information	D/A
E. Referrals to treatment	Subset of those in need (D), referred by the child welfare or FCMH systems to mental health treatment	E/D
F. In treatment	Subset of those referred to treatment (E) who have a treatment start date	F/E
G. Engaged in care	Subset of those in treatment (F) who stay in treatment for at least 6 weeks (based on treatment discharge minus treatment start date)	G/F
H. Continuing care	Subset of those engaged in treatment (G) who are still receiving treatment after 90 days	H/G

^a^N/A: not applicable.

### Digital Health Application (Chorus) and Approach

The FCFN intervention incorporates digital health technology grounded in participatory informatics (using the secure web-based Chorus platform [Chorus Innovations, Inc], which involved end users and navigators in app codevelopment as an inherent and critical component of successful navigation [38]). Chorus is an established digital health platform that provides the basis for a web application named Fostering Connections, which is accessible to a broad range of people (similar to visually creating slides in Microsoft PowerPoint) and includes interactive messaging and mobile web app capabilities [38]. Fostering Connections was adapted based on feedback from CWI families and stakeholders and is designed to support the navigation process across multiple dimensions by (1) supporting bidirectional SMS text messaging (and shared dashboards) to enhance communication between navigators and families, (2) including reminders for upcoming session and behavioral health services appointments, (3) storing navigation documents and resources, and (4) including surveys for completion by navigators. Thus, it is a proven and reliable system that will allow navigators to effectively opt in as well as communicate with, and engage, participants and other providers; in addition, it can be developed and tailored according to research study needs.

### Hypotheses

We hypothesize that youth receiving the FCFN intervention will have increased rates of mental health treatment initiation and engagement compared with youth receiving standard of care. We will also explore hypothesized mechanisms of intervention impact (eg, navigator satisfaction, youth treatment motivation, and perceived barriers to care) as well as potential moderators (eg, sex, race, ethnicity, and out-of-home placement status).

## Methods

### Study Design

Up to 5 FCFNs and 80 families (caregiver-youth dyads) will participate in the RCT to assess the preliminary impact of the FCFN intervention on mental health services use. CWI youth (in in-home or out-of-home placement) aged between 12 and 17 years as well as their caregivers will be referred for study participation at the point of referral from the child welfare system to a partnering mental health clinic (hereinafter referred to as the foster care mental health [FCMH] clinic) that specializes in serving foster care youth by linking them to specialty mental health services. Enrolled families will be randomized to either the FCFN intervention or standard of care. Enrolled CWI youth and their caregivers will complete a computerized set of questionnaires (on a tablet device, mobile phone, PC, or laptop computer) at baseline, midintervention (1-month after baseline), and at 3 and 6 months after randomization.

The trial has been registered with ClinicalTrials.gov (NCT04506437).

### Intervention Condition (FCFN)

**Figure 2:**
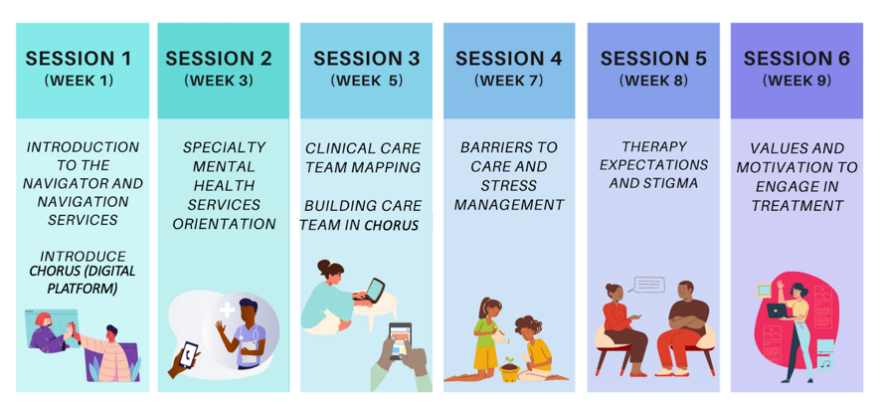
Session-by-session content.

Families (n=40) who are randomized to the FCFN intervention will complete six 60-minute intervention sessions in the first 2 to 3 months of the 6-month period. Sessions can be scheduled on a weekly or biweekly basis. The 6 sessions, described in Figure 2, are as follows: *Introduction to the navigator and navigation services*, *Specialty mental health services orientation*, *Clinical care team mapping*, *Barriers to care and stress management*, *Therapy expectations and stigma*, and *Values and motivation to engage in treatment*. The 6-month intervention period also includes brief check-ins with youth and caregivers, typically conducted through the Fostering Connections web application, by telephone and videoconferencing, depending on the preference and availability of families. The 6 intervention sessions will be audio recorded to separately assess for FCFN intervention fidelity by trained study staff (raters).

### Standard of Care (Clinical Case Management Services) Condition

Families (n=40) who are randomized to standard of care (comparison condition) will receive clinical case management services *as usual*, delivered by FCMH clinic case managers. The role of FCMH clinic case managers is to identify mental health treatment referral resources in the community that match current youth mental health service needs. FCMH clinic case managers facilitate direct referral to a community provider and then provide this information for the family to link to community-based care. FCMH clinic case managers do not typically meet with youth and their families directly but rather follow the existing FCMH clinic protocol of facilitating referrals and linkage via telephone and other communication with youth and their families and their FCMH clinic screening clinician.

### Participants

#### FCMH Clinic Study Sample

Recruitment for the study commenced in August 2020 and will conclude by March 2024. Historically, the FCMH clinic has served an annual average of 100 unduplicated youth aged 12 to 17 years with mental health screening and other related services for those with medical necessity. The average length of time in the court dependency process is 6 months, but can be as long as 12 months in cases of family reunification, and up to 18 months in cases of permanency planning or transition-age youth. Youth who reach the age of 18 years while receiving FCMH services are eligible for, and continue to receive, services without disruption.

#### Racial, Ethnic, and Sex Considerations

FCMH clinic data from the 2017-2018 period suggest that the ethnic representation of youth will be approximately 29% Hispanic or Latinx and that the racial representation will be 29% African American, 3% American Indian or Alaska Native, 18% Asian or Pacific Islander, 14% European-American or White, 3% multiethnic, and 4% other. Of the FCMH clinic adolescent caseload, male adolescents represent approximately 41%, and female adolescents represent approximately 59%. African American and Latinx youth are disproportionately represented, given that, of the total population in the clinic’s surrounding area, African Americans comprise only 6.1% and Latinx only 15.1%.

#### Behavioral Health Considerations

On the basis of FCMH clinic data from the 2017-2018 period, medical records identified the primary disorder diagnosis as 43% adjustment, 24% mood, 22% anxiety (including posttraumatic stress disorder [PTSD]), 7% behavioral (eg, oppositional), 1% psychosis, and 3% deferred.

#### Inclusion and Exclusion Criteria

Eligible participants will be youth aged between 12 and 17 years who have an active dependency petition with the local unified family court (newly filed or existing when the RCT commences), are referred to the FCMH clinic for services, have an involved caregiver or legal guardian who can consent and participate in the intervention, and whose family has mobile phone access. The exclusion criteria include youth and caregivers who are monolingual in languages other than English or Spanish, caregiver impairment that would preclude providing informed consent, and the lack of an available guardian for consent.

#### Recruitment and Consent

Study staff will be embedded in the FCMH clinic setting to introduce the study and procedures to eligible families at the time that families meet with FCMH clinic staff (eg, to complete the mental health screening). FCMH clinic staff will refer eligible families to meet with study staff. Interested youth and families will be separately assented or consented in a private place (FCMH clinic staff will not be involved). We will obtain a waiver of parental consent for referred youth.

#### Randomization

Participants will be randomized (using a computer-generated randomization tool) to either the FCFN intervention group (40/80, 50%) or the standard of care group (40/80, 50%) directly after consent and completion of the baseline assessment.

### Ethics Approval

This study was approved by the institutional review board at the University of California San Francisco (268565) and was conducted in accordance with the ethical standards of the Helsinki Declaration.

### Assessment Data Collection

Multiple sources of data will be collected, including electronic medical records (EMRs); mobile phone or web entry in the Fostering Connections web application; and self-reports from the FCFNs, youth, and caregivers (Textbox 1). Cascade mental health services–level data will be extracted from EMRs at baseline and at 1, 3, and 6 months for participants in both conditions. Assessments will be conducted via mobile phone or tablet device, depending on participant preference.

**Study measures.**

**Identification (percentage of court cohort aged 12-17 years; electronic medical record [EMR])**

- Percentage screened (Child and Adolescent Needs and Strengths [CANS; short form]), percentage assessed (CANS), and percentage in need
- Time to screening, full assessment, and determination of need (number of days)

**Transition (percentage of those identified as in need; EMR)**

- Percentage referred for treatment and percentage who initiated treatment
- Time to referral and initiation (number of days)

**Retention (percentage of those referred for treatment; EMR)**

- Engaged in care (≥6 weeks of attendance)
- Continuing care (number of total sessions completed)

**Navigator background (foster care family navigator [FCFN])**

- Age, race and ethnicity, education, training, and years in child welfare field

**Number and types of contacts with navigator (Chorus platform, FCFN, youth, and caregivers)**

- Video, SMS text messaging, in person, and telephone

**FCFN intervention fidelity (FCFN)**

- Developed as part of prior study procedures

**Therapeutic Working Alliance (youth)**

- Twelve-item Working Alliance Inventory [39], used in the Cannabis Youth Treatment Study [40], that assesses youth’s perception of therapeutic alliance over time

**Navigation Satisfaction Tool, Part I [41] (youth and caregivers)**

- Twelve items that address client satisfaction with navigation services (eg, How satisfied are you with the navigator’s ability to listen and understand your concerns? How satisfied are you with the frequency of contact with the navigator?)

**Evidence-Based Practice Attitude Scale-50 (FCFN)**

- A 50-item scale [42], developed and validated with mental health service providers, that assesses global attitudes toward the adoption of evidence-based practices

**Barriers to Care Questionnaire** **(youth and caregivers)**

- A 39-item scale (subscales of skills, marginalization, stigma, expectations, knowledge and beliefs, and pragmatics) [43] that assesses perceived barriers to child health care access; items are rated on the extent of the problem (ranging from 100 for “no problem” to 0 for “very big problem”), with higher scores indicating fewer barriers (items are at 5.7 grade reading level)

**Motivation for Youth’s Treatment** **Scale** **(youth and caregivers)**

- A psychometrically valid 8-item scale [44] that youth and caregivers complete to measure intrinsic treatment motivation with the subscales of problem recognition and treatment readiness

**Mobile health (mHealth) feasibility or satisfaction (Chorus platform, youth, and caregivers)**

- Mobile phone type, mobile phone service interruptions, number of mobile phone numbers, most preferred types of SMS text messages, number of messages opened or read, and responses

**Usability of mHealth intervention components or “system”: System Usability Scale (SUS; FCFN, youth, and caregivers)**

- A 10-item measure [45] widely used to evaluate web applications that has proven superior to other measures; the SUS yields a single number representing a composite measure of the overall usability of the system being studied, and scores will be compared with established norms for levels of usability; our goal is a score of 80 (top 10% of scores), which reflects excellent usability and is associated with recommending the product to a friend

**Sociodemographics (EMR, youth, and caregivers)**

- Race, ethnicity, sex, education, income, legal history, and placement type

**Pediatric Symptom Checklist (EMR, youth, and caregivers)**

- A 35-item measure of child emotional and behavioral problems that has been identified as a feasible method for the early detection of mental health difficulties [46] and intervention outcomes measurement [47]; items are rated as “never present” (0), “sometimes present” (1), or “often present” (2), with the total (summed) score ranging from 0 to 70; for children and youth aged 6 to 18 years, the cutoff score is 28 (≥28=impaired)

**University of California Los Angeles Posttraumatic Stress Disorder Reaction Index for Diagnostic and Statistical Manual of Mental Disorders, Fifth Edition (DSM-5; youth)**

- An instrument that screens for exposure to traumatic events and the frequency of posttraumatic stress disorder symptom occurrence during the past month (ranging from 0=none of the time to 4=most of the time) [48]; items map directly onto DSM-5 intrusion, avoidance, and arousal criteria, whereas 2 additional items assess associated features (fear of recurrence and trauma-related guilt); scoring algorithms permit tabulation of the total score as well as B, C, and D subscale scores

**Brief Symptom Inventory (caregivers)**

- A 51-item measure of past week mental health symptoms [49]

**Parent-Adolescent General Communication Scale (youth and caregivers)**

- A scale that assesses positive and negative aspects of communication via 2 subscales (open family communication and problems in family communication) [50], with 5 items assessing the frequency of communication topics

**Parental Monitoring Questionnaire (youth and caregivers)**

- A 24-item measure of parental monitoring across 4 areas or subscales (parental knowledge, child disclosure, parental solicitation, and parental control), with higher scores indicating higher levels of monitoring domains [51]

### Measures

#### Overview

Youth and caregivers in both conditions will each complete the electronic study assessment at baseline and at 1, 3, and 6 months (4 times in total). Unless it is specifically noted, all of the following measures will be collected from youth and caregivers across both conditions. FCFN navigator measures are also included.

#### Primary Outcome Measures

##### Cascade of Care Services

We will collect EMR data that will include (1) percentage screened (for mental health needs), percentage assessed (received full mental health assessment), and percentage in need (determined to meet medical necessity criteria for mental health services); (2) time to screening, full assessment, and determination of need (number of days); (3) percentage referred for mental health treatment as well as percentage who initiated treatment; (4) time to referral and initiation (number of days); (5) whether the youth engaged in treatment (whether they had ≥6 weeks of treatment attendance); (6) number of total mental health treatment sessions completed; (7) sociodemographics (to provide collateral EMR data on race and ethnicity, sex, education, income, legal history, and placement type); (8) Child and Adolescent Needs and Strengths (CANS) screener and full assessment (the number and descriptions of needs and strengths and based on the Diagnostic and Statistical Manual of Mental Disorders, Fifth Edition [DSM-5], diagnosis per the CANS rating and resources manual with regard to children and youth aged 5-18 years); and (9) Pediatric Symptom Checklist score (collected by the specialty mental health provider and entered into the EMR as part of standard clinical and billing practice).

##### Therapeutic Working Alliance

The 12-item Working Alliance Inventory (WAI) [39], which was used in the Cannabis Youth Treatment Study [40], assesses youth perception of therapeutic alliance over time.

##### Navigation Satisfaction Tool, Part I

The 12-item Navigation Satisfaction Tool (NAVSAT), Part I [41], addresses participant satisfaction with navigation services (eg, How satisfied are you with the navigator’s ability to listen or understand your concerns? How satisfied are you with the frequency of contact with the navigator?)

##### Barriers to Care Questionnaire

This 39-item scale (subscales of skills, marginalization, stigma, expectations, knowledge and beliefs, and pragmatics) assesses perceived barriers to youth health care access [43]. Items are rated on the extent of the problem (ranging from 100 for “no problem” to 0 for “very big problem”). Higher scores indicate fewer barriers. The items are at 5.7 grade reading level, and thus youth can complete this questionnaire.

##### Motivation for Youth’s Treatment Scale

This is a psychometrically valid 8-item scale that youth and caregivers complete to measure intrinsic treatment motivation with the subscales of problem recognition and treatment readiness [44].

##### System Usability Scale

The System Usability Scale (SUS) [52] is a 10-item measure that is widely used to evaluate web applications and has proven superior to other measures. The SUS yields a single number representing a composite measure of the overall usability of the system being studied [45]. The SUS scores will be compared with established norms for levels of usability. Our goal is to reach a score of 80, which is in the top 10% of scores, reflects excellent usability, and is associated with recommending the product to a friend (youth and caregivers; intervention group only).

##### Pediatric Symptom Checklist

This 35-item measure of child emotional and behavioral problems [46] is widely used in diverse pediatric primary care settings and has been identified as a feasible method for the early detection of mental health difficulties and intervention outcomes measurement [47]. Items are rated as “never present,” “sometimes present,” or “often present” and scored 0, 1, and 2, respectively. The total (summed) score ranges from 0 to 70; for children and youth aged 6 to 18 years, the cutoff score is 28 (≥28=impaired). We will collect this separately and in addition to available EMR Pediatric Symptom Checklist data to measure changes in symptoms over time (ie, for repeated measures).

##### Brief Symptom Inventory

This 51-item measure will be used to rate the extent to which parents have been bothered in the past week by their own mental health symptoms [49].

##### Texas Christian University Drug Screen 5

This 17-item measure assesses past 12-month quantity and frequency of alcohol and drug use and the perceived level of functioning and impairment associated with substance use (youth only) [53].

##### Parent-Adolescent General Communication Scale

This scale will assess the positive and negative aspects of general communication via 2 subscales (open family communication and problems in family communication) [50]. Five items assess the frequency of communication topics.

##### Parental Monitoring Questionnaire

This 24-item measure is designed to assess parental monitoring across 4 areas or subscales (parental knowledge, child disclosure, parental solicitation, and parental control) [51]. Higher scores indicate higher levels of monitoring domains.

##### Child and Adolescent Services Assessment

This assessment consists of a semistructured interview that obtains information about service use for mental health problems across multiple sectors (eg, juvenile legal system and schools) [54]. Information about the type of facility, the professional discipline of provider or providers, outpatient services, and out-of-home placements is obtained, and the measure has adequate reliability (intraclass correlation coefficient=0.74-0.76). We will categorize the length and nature of treatment received to increase the generalizability of our study results. We will also add items related to participants’ arrest history.

##### University of California Los Angeles PTSD Reaction Index for DSM-5

This instrument is used to screen for exposure to traumatic events and assess the frequency of occurrence of PTSD symptoms in the past month [48]. We will use a modified version of this measure that will only ask about the total number of exposures to traumatic events and not the specific type of event or any details related to the event.

##### Sociodemographics

We will collect data on race, ethnicity, sex, education, income, legal history, and placement type.

##### Number and Types of Contacts

Data regarding the number and types of contacts between the FCFNs and youth and caregivers will be collected from the Fostering Connections web application (eg, total number of messages sent and received, number of messages read, content of the messages, and number of contacts with the FCFNs).

##### FCFN Feasibility, Acceptability, and Satisfaction: Evidence-Based Practice Attitude Scale-50

This 50-item scale was developed and validated with mental health service providers and assesses global attitudes toward the adoption of evidence-based practices [42].

##### FCFN Feasibility, Acceptability, and Satisfaction: FCFN Fidelity Forms

As part of intervention delivery, the FCFNs will rate the adherence to sessions (eg, activities completed and number of contacts) of youth and caregivers. The FCFNs will also complete their own feedback session forms after each of the six 60-minute sessions to document the occurrence of planned activities and content and the occurrence of additional activities (that might not have been part of the FCFN manualized intervention), as well as any aberrant events.

##### Exit Interviews With Youth and Caregivers

A subsample of 12 families or 24 participants (6 youths and 6 caregivers or 12 participants from each condition) will be invited to participate in an exit interview at the end of the 6-month intervention period to inform feasibility, acceptability, and satisfaction with the FCFN and standard of care (clinical case management services) conditions. Each participant will be offered a US $40 gift card (applicable to those who are allowed to receive it).

##### Exit Interviews With Foster Care Family Navigators

Up to 5 FCFNs will be invited to participate in an exit interview at the end of the pilot RCT study phase to provide more detailed feedback on their experiences of delivering the FCFN intervention and to inform future trials. Each participant will be offered a US $40 gift card (applicable to those who are allowed to receive it).

### Statistical Analysis

#### Quantitative Analysis

##### Overview

Analyses will be conducted using SAS 9.4 (SAS Institute Inc) [55]. Preliminary analyses will include an examination of descriptive statistics and distributions. Scaled psychometric measures (eg, the Barriers to Care Questionnaire) will be scored according to established algorithms and internal consistency reliability examined. Item-level missing data will be minimized through the programming of surveys; multiple imputation (PROC MI) will be used for item-level missing data if missingness is random. A unified statistical framework—generalized linear mixed models (GLMMs; PROC GLIMMIX)—will be used for most analyses. These models account for the clustering of participants and for unit-level missing data.

##### Cascade Outcomes

GLMM analyses of dichotomous outcomes (screening, treatment initiation, etc) will include intervention (FCFN group vs control group) as a fixed between-participants effect. Cox regression analysis (PROC PHREG) will be used to compare the interventions on time-to-event outcomes (eg, time to assessment) using a shared frailty model that accounts for clustering within pods. The potential mediators and mechanisms of change, measured longitudinally (at baseline and at 1, 3, and 6 months) only for FCFN intervention participants, will be initially examined for intervention impact using GLMMs that include a repeated within-participants time effect. The majority of the mediators are scaled variables, anticipated to have normal distributions, but final models and link functions will be chosen based on observed distributions. It will not be possible to test mediation without data from the control group. Therefore, we will identify potential mediators to be studied in the future trial by first determining which potential mediators change between baseline and month 1 or month 3 and then examining whether month 1 or month 3 values and changes between baseline and month 1 or month 3 are associated with cascade outcomes.

##### Moderators

We will examine potential moderators (eg, sex, gender, race and ethnicity, and mental health symptoms) of intervention impact by adding the potential moderators one at a time, given the modest sample size, to the models used to examine cascade outcomes and mediators. Analyses of cascade outcomes will include moderator × intervention interactions, and analyses of mediators will include moderator × time interactions.

#### Qualitative Analysis

All individual interviews will be digitally recorded and transcribed verbatim. Interview facilitators will review the recording and write notes within 48 hours of each interview completion to construct an executive summary of the main discussion points and topics. Executive summaries have been used successfully in the work conducted by the principal investigator (MTS) to provide data quickly and provide evidence of whether discussion theme saturation is achieved. Key data from executive memos will be immediately entered into ATLAS.ti software (version 7; ATLAS.ti Scientific Software Development GmbH), and the results will be presented to the investigator team. After the completion of the executive summaries and development of the FCFN intervention content, interview data will be analyzed according to inductive thematic analysis methods. An initial codebook will be developed based on the interview guides and corresponding transcripts. To improve reliability and to ensure adequate intercoder agreement, one of the authors (ED) and a clinical research coordinator will compare coding patterns and refine the codebook until consensus is reached and then generate memos to highlight connections between codes and subcodes. Quotations from participants will be compiled and concepts and relationships pertinent to core themes developed. We will seek out and compare unique themes relevant to the analytic subgroups (eg, those based on age, sex, gender, race, and ethnicity) to capture important thematic group differences. Final codes and memos will be compared and combined into overarching themes and subthemes. Themes will be discussed, refined, and named for the final analysis. The principal investigator will provide guidance on coding and analysis at regular study meetings. ATLAS.ti software (version 7) will be used to facilitate the analyses.

## Results

The study was funded by the National Institute of Mental Health on April 20, 2020. The trial is open and recruiting. We propose the enrollment of 80 dyads by March 2024 and final data collection by September 2024. After final data analysis and the writing of the results, the manuscripts will be submitted to appropriate journals for dissemination, with main findings published by March 2025.

## Discussion

### Summary

This study is a protocol for an RCT testing an empirically derived FCFN intervention with adjunctive digital health technology (the Fostering Connections web application) to support linkage to, and engagement in, mental health services among CWI youth. The FCFN intervention encompasses six 60-minute family-based intervention sessions designed to address barriers to, and build motivation for, engagement in outpatient community-based mental health services for CWI youth (aged 12-17 years). On the basis of prior literature on health navigator models delivered in community-based settings, we hypothesize that youth receiving the FCFN intervention will have higher rates of mental health treatment initiation and engagement than youth receiving FCMH clinic standard of care (clinical case management services).

The strengths of this study include the use of a randomized study design to maximize internal validity and scientific rigor as well as the use of empirically supported intervention models (ie, health navigation, family-based support, and motivational interviewing) that have been successfully implemented in community-based settings with diverse populations. The study also builds off prior community-engaged mixed methods work involving interagency collaboration, EMR data collection, and qualitative research (eg, system stakeholder interviews, youth and family interviews, focus groups, and direct observation) to develop and iteratively refine the FCFN intervention and study procedures to enhance scientific rigor and relevance. The study is also designed to maximize real-world implementation potential through the delivery of the FCFN intervention by existing staff at a community-based mental health clinic for CWI youth.

Potential limitations and design considerations include a partnering clinic that does not serve a sufficiently large sample size of CWI youth to be the sole site for the next phase of the large-scale trial. However, our investigators have existing research partnerships with other nearby large northern California counties that would allow us to expand and conduct large-scale FCFN intervention testing with other mental health clinics serving CWI youth using the same design approach as the pilot trial. Furthermore, sample size limitations may limit the current potential to analyze outcomes by those receiving the FCFN intervention as part of in-home services versus out-of-home placement and other demographic variables (eg, ethnoracial identity, gender, and socioeconomic status). Placement type and stability are important to consider in terms of intervention impact; thus, we will descriptively explore how outcomes may differ according to these variables to inform the future larger trial.

### Conclusions

This study intends to move the field forward in several ways. First, it will extend a recently developed services continuum of care framework for legally involved youth with substance use (the juvenile justice behavioral health services cascade framework; JJ-TRIALS) and address the mental health service strengths, gaps, and needs of CWI youth, families, and systems. Second, the pilot trial design will provide an opportunity to engage community stakeholders and leverage existing staff resources for *real-world* intervention testing while also allowing for the measurement and observation of design feasibility and acceptability for a future large-scale hybrid 1 (efficacy or effectiveness) trial. Third and last, the incorporation of digital health tools will aid the navigation process (eg, tracking, care coordination, and communication) to ensure that CWI youth are receiving optimal care that navigators, outside providers, and other relevant CWI systems can measure. The inclusion of an established digital health platform (Chorus for the Fostering Connections web application) supports the navigation process by supporting bidirectional SMS text messaging, appointment reminders, and critical navigation document sharing to enhance communication across FCFNs and families. Taken together, such innovations will allow the study team to provide first-time empirically driven conclusions and recommendations related to the processes, strategies, and preliminary impact of a family mental health navigation model for CWI youth.

## Appendix

Multimedia Appendix 1 Peer-review report by the Mental Health Services Research Committee of the National Institute of Mental Health.

## Abbreviations

ADHD: attention-deficit/hyperactivity disorder
CANS: Child and Adolescent Needs and Strengths
CWI: child welfare–involved
DSM-5: Diagnostic and Statistical Manual of Mental Disorders, Fifth Edition
EMR: electronic medical record
FCFN: foster care family navigator
FCMH: foster care mental health
GLMM: generalized linear mixed model
JJ-TRIALS: Juvenile Justice Translational Research on Interventions for Adolescents in the Legal System
mHealth: mobile health
NAVSAT: Navigation Satisfaction Tool
PTSD: posttraumatic stress disorder
RCT: randomized controlled trial
SUS: System Usability Scale
WAI: Working Alliance Inventory

## Data Availability

Data sharing is not applicable to this study because no data sets have been generated or analyzed yet for the preparation of this protocol manuscript. After analysis and dissemination, data generated and analyzed in the randomized controlled trial will be available from the corresponding author upon reasonable request.
