# Supplementary material for: A Family-Based Mental Health Navigator Intervention for Youth in the Child Welfare System: Protocol for a Randomized Controlled Trial
Source: JMIR Res Protoc. 2023 Sep 12;12:e49999. doi: 10.2196/49999 (PMC10523219; doi:10.2196/49999)
Supplement: Multimedia Appendix 1 [file resprot_v12i1e49999_app1.pdf]

SUMMARY STATEMENT

PROGRAM CONTACT: **(Privileged Communication)** ***Release Date:*** **07/01/2019**

Revised Date:

Application Number: **1 R34 MH119433-01A1**

Principal Investigator

TOLOU-SHAMS, MARINA

Applicant Organization: UNIVERSITY OF CALIFORNIA, SAN FRANCISCO

Review Group: **SERV**
Mental Health Services Research Committee

Meeting Date: **06/11/2019** ***RFA/PA:*** **PAR18-429**
Council: **OCT 2019** ***PCC:*** **82-SECH**
Requested Start: **12/01/2019**

Project Title: **Foster Care Mental Health Family Navigator**

SRG Action: **Impact Score:**
Next Steps: **Visit https://grants.nih.gov/grants/next_steps.htm**
Human Subjects: 30-Human subjects involved - Certified, no SRG concerns
Animal Subjects: 10-No live vertebrate animals involved for competing appl.
Gender: 1A-Both genders, scientifically acceptable
Minority: 1A-Minorities and non-minorities, scientifically acceptable
Age: 1A-Children, Adults, Older Adults, scientifically acceptable

ADMINISTRATIVE BUDGET NOTE: The budget shown is the requested budget and has not been adjusted to reflect any recommendations made by reviewers. If an award is planned, the costs will be calculated by Institute grants management staff based on the recommendations outlined below in the COMMITTEE BUDGET RECOMMENDATIONS section.

1R34MH119433-01A1 TOLOU-SHAMS, MARINA

RESUME AND SUMMARY OF DISCUSSION: In response to PAR-18-429, this R34 resubmission proposes to adapt and test a foster family navigator model for adolescents (ages 12-17) involved in the child welfare system. The application will adapt a navigator framework developed for substance use in justice-involved adolescents (the Juvenile Justice Behavioral Health Services Cascade Framework; JJTRIALS). A mixed methods, stakeholder-engaged approach is proposed for the adaptation process to include mental health outcomes and to develop digital support for coordination of care. The strengths of the application include its focus on a population that is disproportionately at risk for mental health issues and also underserved. Another notable strength of the application is its extremely strong research design, which includes three stages of intervention development and testing, as well as an exceptionally strong collaborations with stakeholders who are highly engaged in the development of digital tools and protocols. In regard to the investigators, the team is well qualified and provides the breadth of complementary areas needed to conduct the proposed research, and they are supported by an outstanding environment. In light of these notable strengths, the application's weaknesses are relatively minor. For example, the application might have more adequately discussed the generalizability of the proposed project, given that strong collaborative relationships are already in place in this setting and may not be in more typical settings. In addition, the application might have more adequately discussed the scope of a future R01 project, and concepts such as feasibility and acceptability could have been more clearly operationalized. Despite these weaknesses, which are readily addressable, this is a highly significant, innovative and well-designed application.

DESCRIPTION (provided by applicant): Youth in the child welfare system have documented high rates of mental health symptoms and experience significant disparities in mental health care services access and engagement relative to youth not in the child welfare system. Navigator models have been developed in the healthcare field to address challenges of service access, fragmentation and continuity that impact quality of care, but at present there is no empirically supported mental health navigator model to address the unique and complex mental health needs of child welfare involved (CWI) youth. This developmental study will be conducted in three phases consistent with study aims. The study will take a mixed-methods, multi-informant participatory research approach to developing, iteratively refining and pilot testing a Foster Care Family Navigator (FCFN) model to improve mental health service outcomes for adolescents (ages 12-17) involved in the child welfare system. The navigator model will leverage digital health technology to engage with and improve care coordination, tracking and monitoring of mental health service needs for these youth and families. The study will adapt the JJ-TRIALS Behavioral Health Services Cascade framework to support a data-driven decision-making approach to improving identification of mental health service needs and outcomes. The study will first utilize a combination of interagency collaborative meetings, youth and family focus groups and qualitative individual interviews with multisystem stakeholders to guide the development of the FCFN protocol. Next an open trial of the 6-month FCFN intervention will be conducted and the protocol iteratively refined through direct participant feedback. The last phase of the study will focus on conducting a modified roll-out design of the FCFN intervention with 75 child welfare involved youth. Three cohorts of 25 youth and caregivers each will receive FCFN services for 6 months and will be compared on primary outcomes of mental health initiation and engagement to 50 youth and caregivers who receive services as usual. We will explore mediators (e.g., satisfaction with navigator, youth treatment motivation, perceived barriers to care) and moderators (e.g., race, ethnicity, sex) of intervention impact to inform intervention mechanisms of change and key demographic and other contextual factors associated with trial outcomes. Finally, we will also conduct qualitative exit interviews with trial participants and navigators to gain a deeper understanding of influences on pilot outcomes that can inform future larger efficacy and effectiveness trials.

PUBLIC HEALTH RELEVANCE

Youth in the child welfare system have documented high rates of mental health symptoms but experience significant disparities in mental health care services access and engagement. System fragmentation, challenges with treatment engagement and poor coordination and continuity of care exacerbate existing disparities. We propose to develop and test a Foster Care Family Navigator intervention that leverages multisystem collaboration and digital health technology to improve child welfare-involved youths' mental health service access, initiation and engagement.

CRITIQUE 1

Significance: 2
Investigator(s): 1
Innovation: 2
Approach: 2
Environment: 1

Overall Impact:
The intention of this study is to use a mixed-methods, multi-informant participatory research approach to develop and refine a Foster Care Family Navigator (FCFN) model to improve mental health service outcomes for adolescents (ages 12-17) involved in the child welfare system. This is a strong, well-written application submitted by an experienced investigative team, and the resubmission is very responsive to the previous reviews. The topic is important and responsive to the funding opportunity announcement. A few minor concerns remain that could benefit from additional clarification, as are detailed below.

1. Significance:
Strengths
- The team has chosen to focus on an important population, namely youth in the child welfare system, given that these youth often experience rates of mental health symptoms and experience significant disparities in mental health care services access and engagement.
- Attention devoted to racial and ethnic minority system-involved youth.

Weaknesses
- None.

2. Investigator(s):
Strengths
- The PI has expertise in mental health, substance use and HIV prevention related research with vulnerable, marginalized populations, as well as several publications and extensive grant experience.
- A more thorough discussion of the role and responsibilities of each co-I well as a discussion of possible overlap of expertise have been added, which strengthen the application.
- Co-I (Shumway) will offer expertise in measurement, statistics, and intervention trials in community settings.

Weaknesses
- None.

3. Innovation:
Strengths
- Use of a dynamic waitlist design.
- Technology assisted navigation.
- Use of existing system and resources.

Weaknesses

- None.

4. Approach:
Strengths
- Use of innovative M Health.
- Building the approach with stakeholders and within an existing system.
- UCSF staff are already embedded into the service setting.
- Using staff members who are already part of the service setting structure (e.g., Pod leaders) is great for integration and sustainability.

Weaknesses
- A discussion has been added about strengths of including staff members who are already part of the service setting structure (e.g., Pod leaders), which is helpful. It also might have been helpful to more fully discuss how generalizable this structure and the role of the pod leaders are to other systems.
- Will an emphasis be placed on referrals to EBPs? There is mention that these services are available in the community? Will that be tracked?

5. Environment:
Strengths
- The environment is outstanding.
- Easy access to a strong network of colleagues.
- Strong letters of support are included.

Weaknesses
- None.

Study Timeline:
Strengths
- Succinct.
- The timeline is ambitious, but now scaled back and reasonable within the timeframe and grant mechanism.

Weaknesses
- None.

Protections for Human Subjects:
Acceptable Risks and/or Adequate Protections
- A clear, detailed plan was provided.

Data and Safety Monitoring Plan (Applicable for Clinical Trials Only):
Acceptable.
- A clear, detailed plan was provided.

Inclusion of Women, Minorities and Children:
- Sex/Gender: Distribution justified scientifically
- Race/Ethnicity: Distribution justified scientifically
- For NIH-Defined Phase III trials, Plans for valid design and analysis:
- Inclusion/Exclusion of Children under 18: Including ages <18; justified scientifically
- A clear, detailed plan is provided. The investigative team has been quite thoughtful about race, ethnicity and gender inclusion details and has confirmed that with their community partners, as is detailed in the application.

Vertebrate Animals:
Not Applicable (No Vertebrate Animals).

Biohazards:
Not Applicable (No Biohazards).

Resubmission:
- The resubmission is responsive to the previous review and is substantially improved.

Applications from Foreign Organizations:
Not Applicable (No Foreign Organizations).

Select Agents:
Not Applicable (No Select Agents).

Resource Sharing Plans:
Acceptable.

Authentication of Key Biological and/or Chemical Resources:
Not Applicable (No Relevant Resources).

CRITIQUE 2

Significance: 2
Investigator(s): 2
Innovation: 1
Approach: 4
Environment: 1

Overall Impact:
This is a resubmission of an R34 application responding to PAR-18-429, calling for pilot studies to develop and pilot test the effectiveness and implementation of family navigator models to increase early access, engagement, and coordination of mental health services for children. The application proposes to develop, with extensive stakeholder engagement, the Foster Care Family Navigator (FCFN) model, targeting the population of youth and caregivers who are child welfare involved (CWI). This population is disproportionately at risk for mental health issues and also underserved. The proposed study has many strengths. It addresses a significant need, involves a team of investigators with relevant and complementary expertise, is innovative in multiple ways (including the integration of a digital health platform as part of the FCFN model), proceeds through three stages of intervention development and testing, specifies hypothesized mechanisms of change and their measures, and benefits from an exceptionally strong academic environment and collaborations with stakeholders across systems. A moderate concern exists regarding the timing and placement of the FCFN model within the unit responsible for conducting mental health screenings of referred CWI youth and caregivers; if youth and caregivers present for screening, it seems that they would have already negotiated many of the barriers to services that navigation models are intended to overcome. Additionally, given the strong collaborations and processes already in place in the systems involved in this study, it is not fully clear that the FCFN model would be easily implementable in “typical” settings. Finally, the application does not adequately describe the scope and purpose of the subsequent large-scale trial that this pilot mechanism is intended to prepare for; the third aim of the pilot is described as testing effectiveness and appears to be designed and powered for that purpose.

1. Significance:
Strengths

- Mental health needs are elevated and unmet in the CWI population.
- Multi-system stakeholder engagement in intervention development is a major strength – family courts, child welfare, public mental health, schools.
- Racial, ethnic, and socioeconomic disparities are recognized and addressed by focusing on the CWI population.

Weaknesses

- FCMH relies on child welfare caseworkers to make referrals for mental health screening and assessment. Once youth/caregivers are seen at FCMH, they have already overcome many of the barriers to accessing mental health services. It seems that this point in the process might be late for a family navigator intervention if early identification and engagement in mental health services are the goals.
- Even with the CA laws and the system for referrals in place, only 28% of CWI in San Francisco were assessed within 2 months of case opening, according to the local data cited in the application. Was this a capacity issue, a referral issue, a youth/caregiver follow-up issue? The reason for this is not clearly explained in the application. The point(s) at which the breakdown occurs, resulting in this low rate, should be clear before a new role is added to an already complex system.
- It is not fully clear whether the pod structure and functions at FCMH constitute a "typical" service setting with typically available resources and personnel. The review criteria for this FOA emphasize that the developed intervention/program should be implementable in typical settings with typical resources and personnel – it is difficult to assess whether the pod directors serving as navigators would be available in other settings, whether this role would be compatible with similar positions in other settings, etc.
- Not many details appear to be provided in regard to the larger trial for which the proposed study serves as a pilot.

2. Investigator(s):

Strengths

- The PI is a clinical psychologist with extensive experience with intervention research in vulnerable populations (mostly juvenile justice), family-based interventions, and trauma.
- Co-I Shumway is a quantitative psychologist with expertise in statistics and measurement.
- Co-I Dauria is a behavioral scientist with expertise in qualitative methods.
- Co-I Borsari is a clinical psychologist with expertise in motivational interviewing.
- Co-I/Site PI Berrick (at UC Berkeley) has extensive child welfare expertise with a focus on foster care.
- Co-I Aguilera (at UC Berkeley) is a clinical psychologist with expertise in digital health and the HealthySMS text messaging system proposed for use in this study.

Weaknesses

- This is a large team of investigators, several of whom have very limited effort devoted to this project and two of whom are at a different institution.
- There appears to be overlap in expertise among several co-Is with little attention to describing the communication/coordination among members of the team; the study team overview reiterates team members' areas of expertise but does not fully clarify how the group will function.
- Borsari's biosketch references a different study.

3. Innovation:

Strengths

- JJ-TRIALS Cascade framework is applied to a new context/population.
- Integration of mHealth/texting/dashboard components into the navigation model is innovative.

- There is potential for particularly innovative aspects of the digital components to be used (e.g., geo fencing), depending on target population and stakeholder feedback.

Weaknesses

- A hybrid type 1 trial does not typically focus on efficacy/effectiveness as stated in the application, but on effectiveness/implementation.

4. Approach:

Strengths

- 3-stage intervention development approach (development, implementation/refinement, testing) is a strength.
- Figure 4 depicts how the FCFN model is hypothesized to affect mental health services outcomes through specific mechanisms of change. Outcome measures and measures of mechanisms are specified.
- Dynamic wait-listed design is a strength.
- Stakeholders are incorporated into each study phase, increasing the chances of sustainability and progress to a subsequent large-scale effectiveness trial.

Weaknesses

- Feasibility and acceptability could have been more clearly operationalized; quantitative measures and targets/benchmarks could be specified up front, in addition to the proposed qualitative exit interviews.
- Using discharge date and assessment date to calculate engagement might be problematic if discharges (as in many service settings) do not occur immediately after clients disengage from services.
- It might have been helpful to explain how the team will integrate what is already known about feasible and valid fidelity measurement approaches into the development of a fidelity assessment.
- A missing part of the Cascade seems to be: of those referred for screening, how many were screened? This is linked to the comment under Significance regarding the context and processes underlying only 28% of newly opened cases being screened by 2 months after case opening and the lack of clarity regarding whether referrals are not made or whether referrals are made but screenings do not occur.
- Even at the intervention development phase, preparation for a future trial could have been strengthened by using an implementation science framework to guide assessment of factors which might impact the use and effectiveness of the FCFN model; domains such as intervention characteristics and interventionist characteristics are already touched on in the planned measures, and characteristics of the inner and outer setting could be added relatively easily.
- In regard to feasibility of achieving the proposed aims in the timeline, this mechanism is intended to support pilot trials for larger effectiveness studies. The proposed sample size and analyses for Aim 3 appear more consistent with an effectiveness trial than a pilot.

5. Environment:

Strengths

- Excellent research environments at both institutions.
- Clear support from partnering agencies and stakeholders in the community – strong letters provided.

Weaknesses

- None noted.

Study Timeline:

Strengths

- Clear.

Weaknesses
- Likely ambitious.

Protections for Human Subjects:
Acceptable Risks and/or Adequate Protections
- Very comprehensive and clear; kudos for a very thoughtful and detailed HSP section.
- Recommend that navigators should complete human subject's protection training; although they are participants themselves, they will be in contact with youth/caregiver participants.

Data and Safety Monitoring Plan (Applicable for Clinical Trials Only):
Acceptable.
- Detailed and appropriate.

Inclusion Plans:
- Sex/Gender: Distribution justified scientifically
- Race/Ethnicity: Distribution justified scientifically
- For NIH-Defined Phase III trials, Plans for valid design and analysis: Not applicable
- Inclusion/Exclusion Based on Age: Distribution justified scientifically
- Appropriate and scientifically justified.

Vertebrate Animals:
Not Applicable (No Vertebrate Animals).

Biohazards:
Not Applicable (No Biohazards).

Resubmission:
- The application is moderately responsive to prior reviews: justification of using the CANS as a screening tool is acceptable; transition from juvenile justice to child welfare population is explained; some increase in investigator effort was apparently made. The response to a reviewer concern regarding disentangling of the effects of various aspects of the intervention (i.e., navigator, MI, and mHealth) is adequate. The issue of generalizability beyond California (due to laws and systems in place in that state) is somewhat addressed, but other generalizabilty concerns remain (i.e., FCMH as the source of youth/caregiver participants will likely yield a sample more likely to have engaged in mental health screening, assessment, and treatment). Insufficient response is provided in regard to the need for a communication/coordination plan, given the number and locations of investigators.

Applications from Foreign Organizations:
Not Applicable (No Foreign Organizations).

Select Agents:
Not Applicable (No Select Agents).

Resource Sharing Plans:
Acceptable.

Authentication of Key Biological and/or Chemical Resources:
Not Applicable (No Relevant Resources).

CRITIQUE 3

Significance: 1
Investigator(s): 1
Innovation: 1
Approach: 2
Environment: 1

Overall Impact:
This is a significant R34 application that focuses on addressing mental health needs of youth in the child welfare system. To date, there is no evidence supporting the use navigator models to address the unique and complex mental health needs of child welfare involved (CWI) youth. This R34 study would take steps to address this gap. The study will iteratively refine and pilot test a Foster Care Family Navigator (FCFN) model to improve mental health service outcomes for adolescents (ages 12-17) involved in the child welfare system. The model leverages digital health technology (and a data-driven decision-making approach) and stakeholder engagement to develop technology and protocols that aim to improve care coordination, tracking and monitoring of mental health service needs for these youth and families. What is developed is then pilot-tested in the final study aim. This work lays the foundation for a larger effectiveness trial. The Introduction to this Resubmission responds to prior concerns in a clear manner. The revisions made to the application strengthen this study and address the weaknesses that previously limited enthusiasm for a highly significant study.

1. Significance:
Strengths
- Strong. Research is needed in this area.

Weaknesses
- [None noted]

2. Investigator(s):
Strengths
- The investigative team is strong. Changes to FTE for the PI and the tech developer strengthen the investigative team and lend confidence that they have the expertise and time to carry out study activities.

Weaknesses
- [None noted]

3. Innovation:
Strengths
- This is a highly innovative study.

Weaknesses
- [None noted]

4. Approach:
Strengths
- The approach is well described and well developed.
- Change to the timelines (and the reduction in sample size) make excellent sense, given that this is a pilot study.
- The revisions made to the evaluation plan and, in particular, how participants will be selected for interviews to assess feasibility and acceptability of the intervention improve the design.

Weaknesses
- [None noted]

5. Environment:
Strengths

- The environment is strong.

Weaknesses

- [None noted]

Study Timeline:
Strengths

- The revisions to the study timeline make it more likely that this work can be accomplished in the time specified.

Weaknesses

- [None noted]

Protections for Human Subjects:
Acceptable Risks and/or Adequate Protections
Data and Safety Monitoring Plan (Applicable for Clinical Trials Only):
Acceptable.

Inclusion Plans:

- Sex/Gender: Distribution justified scientifically
- Race/Ethnicity: Distribution justified scientifically
- For NIH-Defined Phase III trials, Plans for valid design and analysis:
- Inclusion/Exclusion Based on Age: Distribution justified scientifically

Vertebrate Animals:
Not Applicable (No Vertebrate Animals).

Biohazards:
Not Applicable (No Biohazards).

Resubmission:

- This resubmission is highly responsive to the previous review.

Applications from Foreign Organizations:
Not Applicable (No Foreign Organizations).

Select Agents:
Not Applicable (No Select Agents).

Resource Sharing Plans:
Not Applicable (No Relevant Resources).

Authentication of Key Biological and/or Chemical Resources:
Not Applicable (No Relevant Resources).

CRITIQUE 4

Overall Impact:
This is a thoughtful and strong application with goals that will improve clinical practice for care of a vulnerable population. All concerns noted in the original application were thoroughly addressed in this application. Given the high rates of disparities in youth in the CWI receiving mental health care as compared to youth not in CWI receiving mental health care, this is a critical area of need and one that should be addressed. The navigator model that is proposed by the investigator indicates the

importance of coordinated and collaborative interagency work. Given the success of navigator models in other populations such as cancer, the foster care navigator is co-develops intervention with other important agencies involved with these patients. They added another layer of engagement by incorporating state of the art mobile health technology. The chances of successful outcomes are strengthened by conducting participatory research with all key stakeholders. Since our existing mental health navigation models do not take into consideration systems-involved youth, the proposal of co-developing the navigator approach with interagency collaboration has the potential to positively change treatment and understanding of mental health conditions in foster care youth.

The study procedures are safe and tolerable without being burdensome especially with the incorporation of m-health. One of the most important strengths of this proposed study is the use of stakeholder engagement throughout the study. The focus groups with CWI youth and families are a strong example of this. In addition, meeting the foster youth and families and agency stakeholder members to a schedule that is flexible to them allows for greater rates of recruitment and retention. The recruitment and retention strategies the investigator proposes are reasonable. In summary, this is a strong resubmission that includes strong stakeholder engagement to test a navigator model for foster youth that if successful will make a major contribution to the mental health care of this vulnerable population.

Protections for Human Subjects:
Acceptable Risks and/or Adequate Protections
Data and Safety Monitoring Plan (Applicable for Clinical Trials Only):
Acceptable.

Inclusion Plans:
- Sex/Gender: Distribution justified scientifically
- Race/Ethnicity: Distribution justified scientifically
- For NIH-Defined Phase III trials, Plans for valid design and analysis:
- Inclusion/Exclusion Based on Age: Distribution justified scientifically

THE FOLLOWING SECTIONS WERE PREPARED BY THE SCIENTIFIC REVIEW OFFICER TO SUMMARIZE THE OUTCOME OF DISCUSSIONS OF THE REVIEW COMMITTEE, OR REVIEWERS' WRITTEN CRITIQUES, ON THE FOLLOWING ISSUES:

PROTECTION OF HUMAN SUBJECTS: ACCEPTABLE

INCLUSION OF WOMEN PLAN: ACCEPTABLE

INCLUSION OF MINORITIES PLAN: ACCEPTABLE

INCLUSION OF CHILDREN PLAN: ACCEPTABLE. Adolescents (age 12-17) involved in foster care and the juvenile justice system are the focus of the application.

COMMITTEE BUDGET RECOMMENDATIONS: The budget was recommended as requested.

Footnotes for 1 R34 MH119433-01A1; PI Name: TOLOU-SHAMS, MARINA

NIH has modified its policy regarding the receipt of resubmissions (amended applications). See Guide Notice NOT-OD-14-074 at http://grants.nih.gov/grants/guide/notice-files/NOT-OD-

14-074.html. The impact/priority score is calculated after discussion of an application by averaging the overall scores (1-9) given by all voting reviewers on the committee and multiplying by 10. The criterion scores are submitted prior to the meeting by the individual reviewers assigned to an application, and are not discussed specifically at the review meeting or calculated into the overall impact score. Some applications also receive a percentile ranking. For details on the review process, see http://grants.nih.gov/grants/peer_review_process.htm#scoring.
